# Involvement of silver and palladium with red peanuts skin extract for cotton functionalization

**DOI:** 10.1038/s41598-023-43267-8

**Published:** 2023-09-26

**Authors:** Hossam E. Emam, Nancy S. El-Hawary, Hamada M. Mashaly, Hanan B. Ahmed

**Affiliations:** 1 Pre-treatment and Finishing of Cellulosic Fibers, Textile Research and Technology Institute, National Research Centre, 33 EL Buhouth St., Dokki, Giza, 12311 Egypt; 2 Dyeing, Printing and Auxiliaries Department, Textile Research and Technology Institute, National Research Centre, 33 EL Buhouth St., Dokki, Giza, 12311 Egypt; 3 Chemistry Department, Faculty of Science, Helwan University, Ain-Helwan, Cairo, 11795 Egypt

**Keywords:** Nanoscale materials, Environmental chemistry

## Abstract

A systematic study is currently demonstrated approach for approving the superior role of silver and palladium metallic particles in acting the role of mordant with acquiring the dyed cotton fabrics excellence in color fastness with additional functions of antimicrobial potentiality and UV-protection action. Whereas, samples were dyed with extract of red peanuts skin as natural textile colorant (RPN dye). The represented data revealed that, in absence of mordant, the samples treated with metal precursors prior to dyeing were exhibited with the excellent color strength, color fastness, antimicrobial action and UV-protection action. Color fastness (washing, rubbing and light fastness) was estimated to be in the range of very good–excellent. Sample pretreated with silver salt and dyed in the absence of mordant was graded with excellent UV-protection action (UPF 31.5, UVB T% 2.6% and UVB blocking percent 97.4%). Antimicrobial potency against *E. coli*, *S. aureus* and *Candida albicans* through inhibition zone and the reduction percent was approved to be in the range of excellence (93.01–99.51%) for the samples dyed in absence of mordant and pretreated with either silver or palladium precursors.

## Introduction

Natural dyes are superiorly exhibited with biocompatibility and wide applicability in various pharmacological purposes. Compared to their synthetic equivalents, they are less harmful in terms of environmental safety and health. Annatto, turmeric, and saffron are examples of natural dyes that are widely applicable as food additives. However, metallic mordants are mainly required as they are disadvantageous with poor fastness, due to they are weakly bonded to the textile materials^1,2^.

On the other hand, protective textiles could be identified as clothing materials that could successively protect the customer body from any of external threating agents such as biological agents, bullet and chemical agent, firing, coldness, and heating. The protective textile materials could cover at least thirty percent of the whole body that could be exploited in sporting, military, and industrial purposes^3–8^.

Cotton fabrics were widely applied in different purposes attributing to their outstanding characters such as, absorptivity, biodegradability, smoothness, and breathability. However, it is disadvantageously characterized with poor ultraviolet shielding, and high flammability and prone, meaning it could be attacked with different microbial strains specially the bacterial strains^9–11^. Numerous researches were reported different investigative techniques for acquiring different textile materials additional functions, like ultraviolet shielding^12–15^, microbicide activities^15,16^, coloration^17,18^, optical activity^19^, insect repellency^20,21^ and self-cleaning^19,22,23^. Moreover, the protective masking and air-filtering textile materials were prepared for protection from chemical warfare gas^24^.

For manufacturing of functional textile materials, numerous researching approaches were considered with the successive application of different organic reagents, like, Triclosan (for bactericidal potency), Benzophenones (for ultraviolet resistance), Dimethylol-dihydroxy-ethylene urea (for wrinkle resistance), Fluorocarbons (for hydrophobic character), long-chain hydrocarbons and Polydimethylsiloxanes (for softening and flexibility)^25,26^. Butane tetra-carboxylic acid, Citric acid and Maleic acid^27–29^ were also applied for preparing cotton fabrics with wrinkle resistance affinity.

In the represented approach, the point of novelty is the demonstration of a systematic study for validation the superior role of silver and palladium particles in enhancing the color fastness and acquiring the dyed cotton fabrics excellent color fastness, antimicrobial potentiality and UV-protective affinity. Whereas, the prepared cotton samples were dyed with extract of red peanuts skin as an example of natural textile colorant^30–32^. Regulative uploading of palladium and silver was proceeded via immobilization within the polymeric matrix of cotton fabrics dyed with the extract of red peanuts skin, whereas, the effect of reaction conditions and reaction sequencing were systematically studied.

## Experimental

### Materials and chemicals

Silver nitrate (AgNO_3_,  ≥ 99.0%, Sigma-Aldrich), palladium chloride (PdCl_2_, 99.0%, Sigma-Aldrich), aluminium sulphate (Al_2_(SO_4_)_3_, Alum, 99.99%, Sigma-Aldrich) and sodium carbonate (Na_2_CO_3_, > 99.5%, Sigma-Aldrich) were exploited as received. Red peanuts were supported from the local market in Egypt. Bleached 100% cotton fabrics (160 g/m^2^) were provided by El-Mahalla Company for Spinning and Weaving, El-Mahalla El-Kubra – Egypt. For cleaning the prepared fabric samples, fabrics (1/50 material: liquor ratio) were washing using 2 g/L nonionic detergent (Hospatal CV – Clariant) at 50 °C for 30 min. Fabrics were rinsed with tap water at room temperature and dried in the atmospheric air.

### Procedure

In the current approach, successive immobilization of silver and palladium particles within dyed cotton fabrics, in order to contribute in fabric coloration and functionalization, was performed. The experimental work could be clarified (Table 1) in the following points;

a- Dyeing with red peanuts in the presence of mordant (sample A): cotton fabrics were dyed with solution of natural dye extracted from red peanuts (5g dispersed in 100 ml of distilled water were boiled for 1 h) at 80 °C, pH 9.5, L: R 1:40 for 1 h. Then for mordanting, the sample was soaked in a solution containing 1.8 g/L of (Alum), pH 7, L: R 1:40, at 80 °C for 30 min.
b- Treatment process (samples B1, B2, C1 & C2): cotton fabrics were treated only with metal precursors, whereas, the fabrics were immersed within solution containing 100 ppm of AgNO_3_ or PdCl_2_ at 80 °C or 60 °C for 30 min, respectively.
c- Post-treatment process (D1 & D2): all the samples were dyed with natural dye then treated with metal precursors.
d- Pre-treatment process (samples E1 & E2): cotton fabrics were immersed within solution containing 100 ppm of AgNO_3_ or PdCl_2_ at 60 °C for 30 min then let the sample dry to be sequentially dyed with natural dye.
e- Pre-treatment in the presence of mordant (F1 & F2): samples were treated with metal precursors then dyed with natural dye to be sequentially treated with mordant (Alum)
f- Simultaneous treatment (G1 & G2): samples were concurrently dyed and treated with metal precursors.

**Table 1:** Description and details for the functionalized cotton samples.

Code	Ag (mg/L)	Pd (mg/L)	Process
A	0	0	Dyeing with penuts then addition of Alum as mordant
B1	100	0	Treatment with Ag^1+^ and Pd^2+^ at 80 °C for 30 min
B2	0	100	
C1	100	0	Treatment with Ag^1+^ and Pd^2+^ at 60 °C for 30 min
C2	0	100	
D1	100	0	Post treatment: dyeing then treatment with Ag^1+^ and Pd^2+^ at 80 °C for 30 min
D2	0	100	
E1	100	0	Pre-treatment: Treatment with Ag^1+^ and Pd^2+^ at 80 °C for 30 min then dyeing
E2	0	100	
F1	100	0	Pre-treatment: Treatment with Ag^1+^ and Pd^2+^ at 80 °C for 30 min then dyeing then treated with mordant (Alum)
F2	0	100	
G1	100	0	Simultaneous process: Treatment with Ag^1+^ and Pd^2+^ at 80 °C for 30 min and dyeing together in the same bath
G2	0	100	

### Analysis and characterization

Native and the currently prepared functional fabrics were both analyzed in order to give information about the change in the topography after fabric treatment with high resolution scanning electron microscopy (SEM Quanta FEG 250 with field emission gun, FEI Company – Netherlands). The chemical characterization of fabrics surface was identified with energy dispersive X-ray spectroscopic (EDX) analysis unit (EDAX AMETEK analyzer) connected to the electron microscopy. X-ray diffraction data for both of pristine and treated cotton fabrics at room temperature with diffractometer (X’Pert MPP with a type PW 3050⁄10 goniometer), whereas, the diffraction angle (2θ) was estimated in range of 10°–80° using monochromatized software of PROFIT (Mo Kα X-radiation at 40 kV, 50 mA and λ = 0.70930 Å) with step size of 0.03°.

Infrared spectral data were detected for cotton fabrics before and after treatment using attenuated total reflection–Fourier Transformation infrared spectroscope (ATR-FTIR). Fabrics were analysed with high resolution JASCO FT/IR–4700 spectroscopy (JASCO Analytical Instruments, Easton, USA) attached to deuterated triglycine sulfate (TGS) detector and conducted with ATR in the infrared range. Spectral data were estimated in wavenumber range of 4000–500 cm^–1^ (using transmission mode (T%), resolution of 4 cm^–1^ with 2 mm/sec scanning speed and 1.0 cm^–1^ interval scanning using 64 repetitious scans average). X-ray photoelectron spectroscopic (XPS) analysis were performed for the samples prepared via pre-treatment process (E1 & E2), using X-Ray–FG ON (400 µm). Whereas, these conditions were concerned; samples were neutralized via low energy electrons (0.1 eV), with hybrid mode (with electrostatic and magnetic lenses), 10–22 scanning and CAE 50 were used and the excitation of photoelectrons by monochromatic Alpha irradiation.

Color coordinates (L, a*, b*) of cotton fabrics before and after treatment were evaluated with colorimeter with (UltraScan Pro, Hunter Lab, USA), pulsed xenon lamps as light source, 10° observer (with D65 illuminant, d/2 viewing geometry and measurement area of 2 mm). L is referring to lightness (0–100) from black to white and a^*^ (+ /–) is for a red/ green ratio, whereas, b^*^ (+ /–) is corresponding to yellow/blue ratio^33^. Moreover, color strength (K/S) was estimated at wavelength of 535 nm and absorption was detected in the range of 350–650 nm. All estimated values were detected for two times and the mean average was taken in account. Colour fastness were examined for the treated fabrics according to the method reported in literature^34^. Washing and rubbing fastness were all tested in accordance to ISO 105-C02, ISO 105-X12 and ISO 105-E04^35^, respectively. While the light fastness was determined using ISO 105-B02 via Blue-scale^36^.

Antimicrobial potency of the treated fabrics against (*Staphylococcus aureus*), gram-negative bacteria species (*Escherichia coli*) and fungal species (*Candida albicans*) was tested via the qualitative methodology of inhibition zone (disk diffusion test)^37^. In this method, the different tested microbial strains were allowed to grow in media for preparing the microbial suspension. 100 μL of microbial colloidal solution was dispersed on agar plates for broth in which it was maintained. The treated fabrics (0.5 cm) were added in the middle of plate to be incubated at 37 °C for one day. The diameter of inhibition zone was estimated in millimeters with slipping calipers in accordance to NCCLS, 1997^37^. After incubating, colony forming units (CFU) were counted for each prepared plate^37,38^.

## Results and discussion

Production of protective textiles becomes more extensively considered for specialized work environments in which there is a risk of exposing to ultraviolet light, fires, chemicals and pathogens, such like in hospitals, police stations, fire departments and battlefields. In the represented approach, a systematic study is demonstrated for approving the superior role of silver and palladium metallic particles in acquiring the dyed cotton fabrics excellent color fastness, microbicidal potentiality and UV-protective affinity. Whereas, the prepared cotton samples were dyed with extract of red peanuts skin (RPN dye) as an example of natural textile colorants.

**Figure 1:**
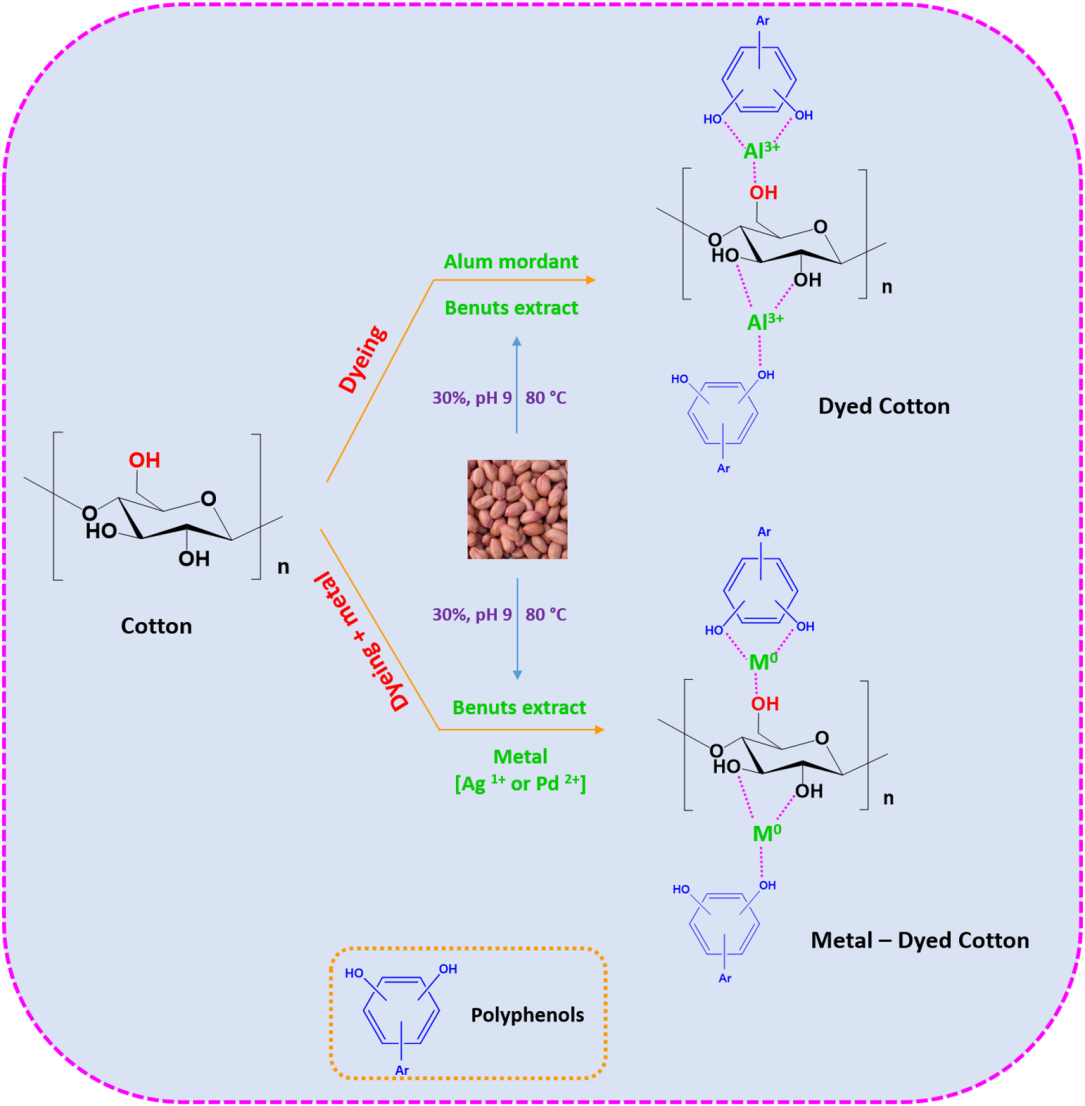
Schematic for formation of dyed cotton and incorporation of Ag & Pd within the dyed cotton fabrics.

According to Fig. 1 that represents a suggestive mechanism for the interaction between cotton fabric, RPN dye and metal precursors, so as it could be declared that, RPN dye is well known as a water-soluble colorant with cationic quaternary ammonium group^39–41^, could interact with the hydroxyl groups in the cellulosic backbone of fabric. Whereas, addition of mordant results in formation of a coordinative complex between RPN macromolecules and Alum particles, in order to enhance the color fastness of the treated fabrics. In the current approach, silver or palladium salt precursors were exploited instead of mordant, as it could form a chemically stable complex with the phenolic groups of RPN dye moieties for concurrent improvement the color fastness and acquire the treated fabric the superior characteristics of either silver or palladium particles for antimicrobial & UV-blocking actions.

### SEM & EDX images

**Figure 2:**
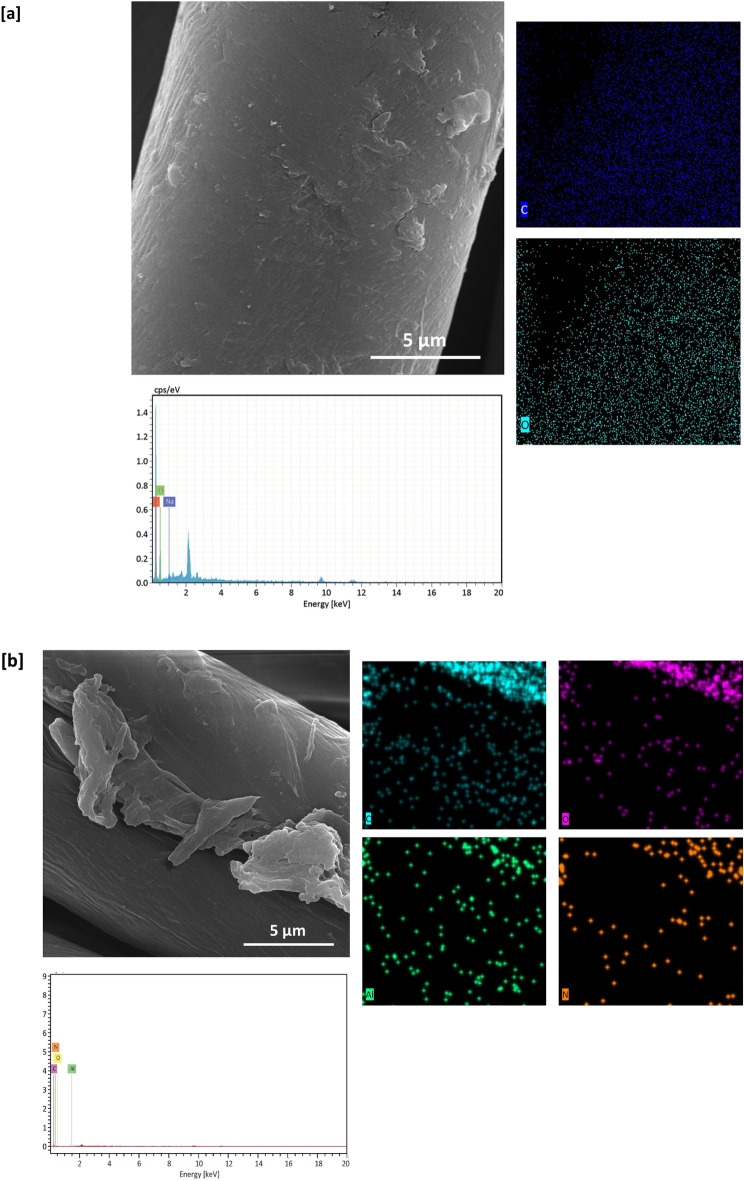

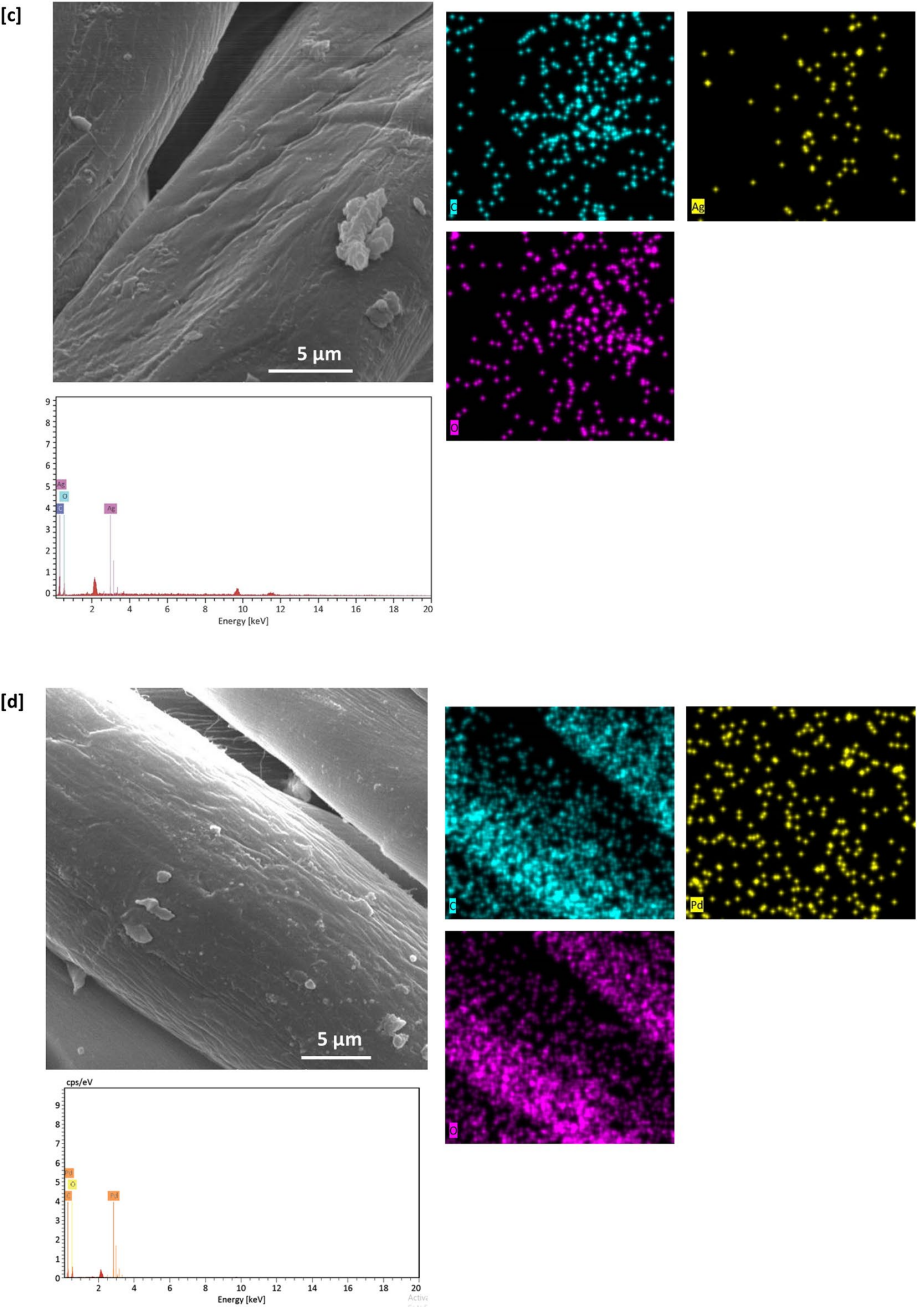

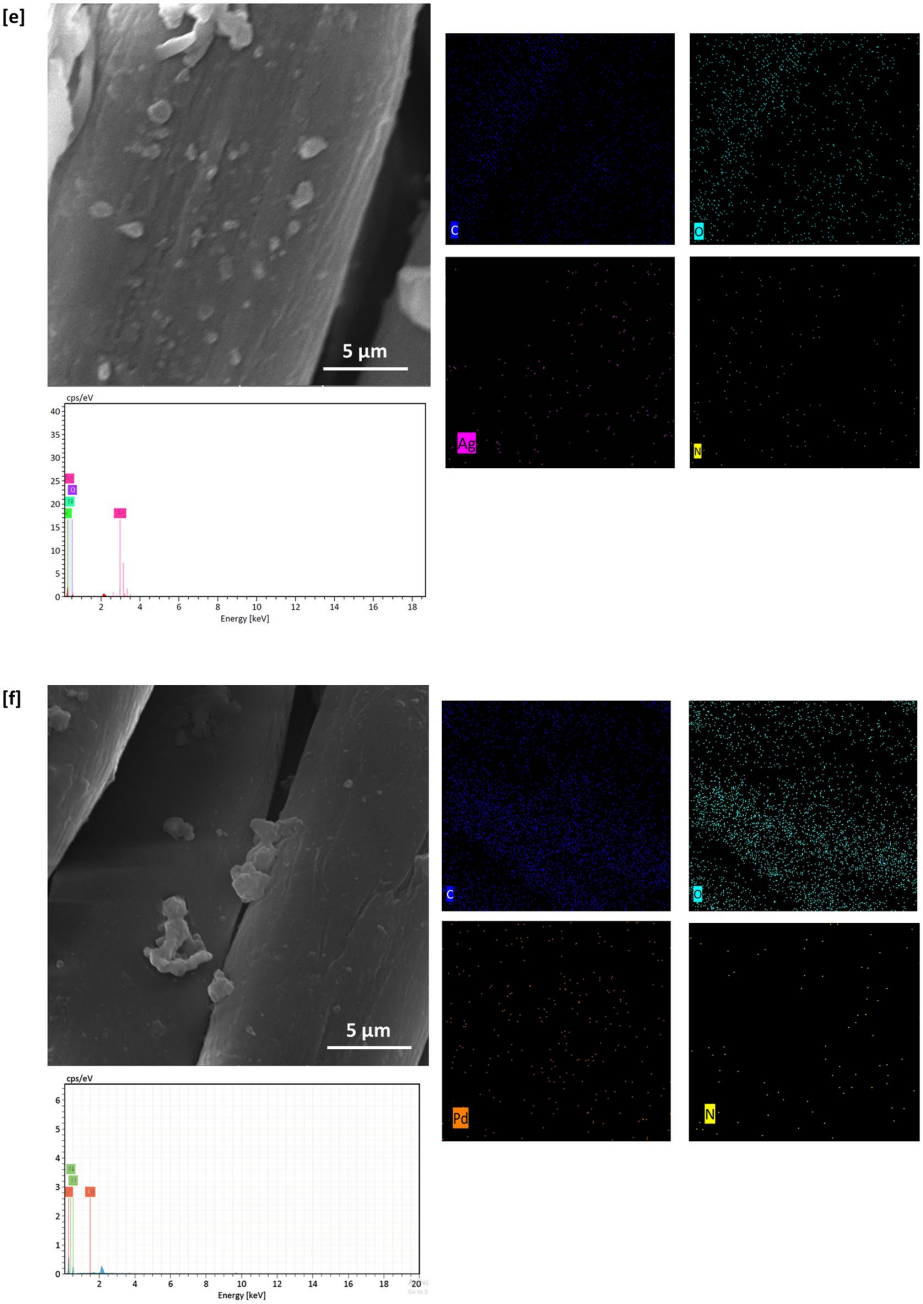
SEM images, EDX and mapping analysis for the modified cotton fabrics; (**a**) untreated, (**b**) A, (**c**) B1, (**d**) B2, (**e**) E1, and (**f**) E2.

SEM & EDX characterizations were performed for both of untreated and treated cotton fabrics to show their morphological and geometrical features and to observe the topographical changes of the examined fabrics after treatment. SEM micrographs were represented in Fig. 2. Figure 2a shows that, untreated cotton fabric was shown with a clear and smooth surface, whereas, Fig. 2b views a well dispersion of RPN dye macromolecules on the surface of dyed fabric (sample A). Figure 2c and d shows that, metal particles were visibly shown on the surface of the treated fabric, this could affirm the successive immobilization of metal particles within fabric matrix.

Figure 2e and f represents that, sequential dyeing of silver-treated fabric resulted in observing more of dispersed particles on the surface of fabric, attributing to the higher affinity of RPN moieties in coordinative bonding with higher amounts of metal particles. These could give an information about the role of RPN dye moieties in regular implantation of well-assembled metal particles. However, lesser amounts of palladium particles were observed by comparing to silver, that, could be attributed to the masking effect of RPN dye macromolecules^42^. Further improvement for successive implantation of metal particles within the undyed and dyed cotton fabrics was achieved via analyzing EDX data, where, Ag & Pd signals were precisely estimated for the treated fabrics (Fig. 2c–f).

### FTIR

**Figure 3:**
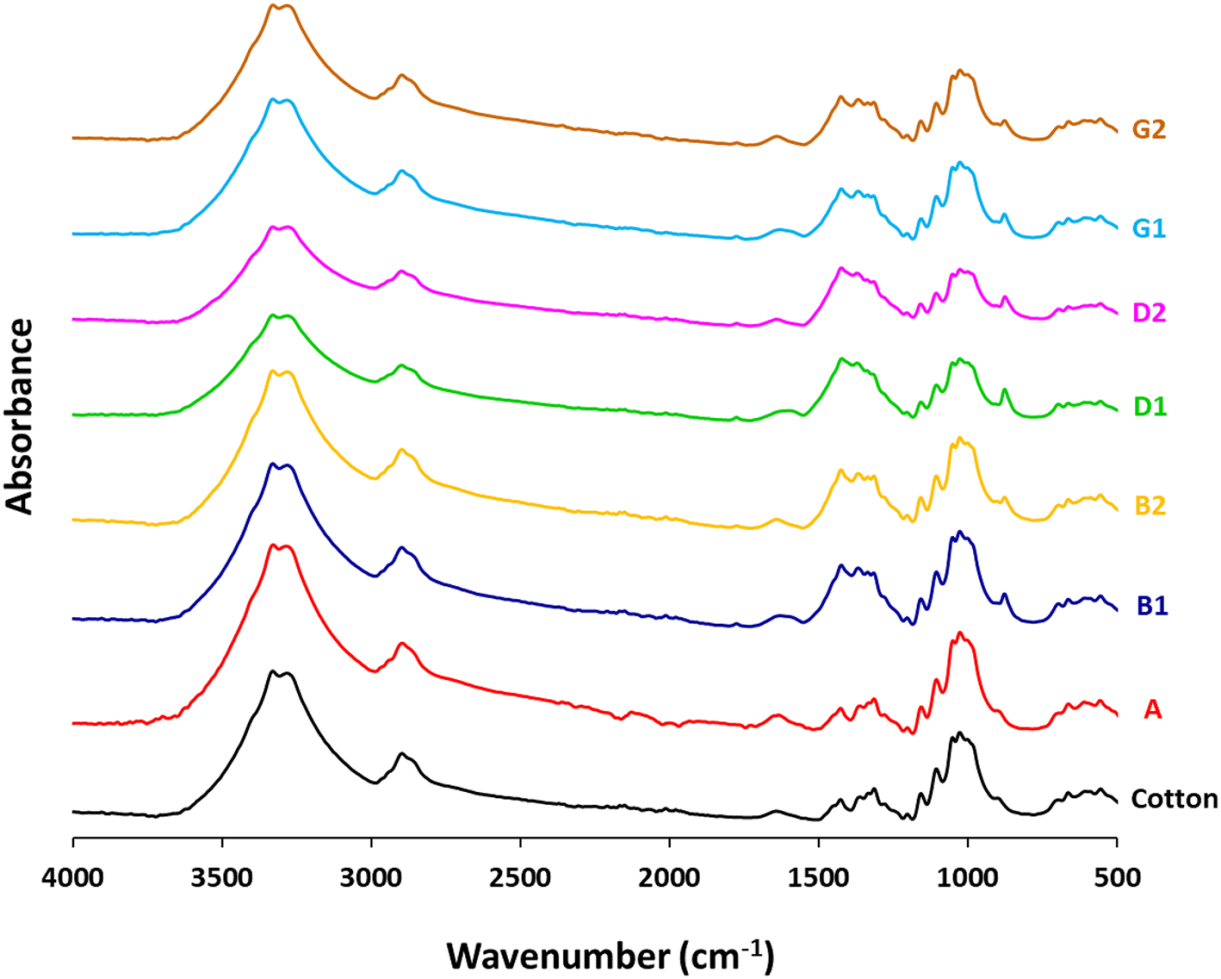
Infrared spectra for the modified cotton fabrics.

FTIR spectra of untreated and treated samples were shown in Fig. 3. Pristine cotton was displayed with three characteristic peaks at 1635 cm^–1^, 2882 cm^–1^ and 3304 cm^–1^, corresponding to C=O, C–H asymmetric stretching and O–H stretching, respectively^43–45^. Transmission peaks of C–O stretching were detected at 1021 and 1152 cm^−1^. Three bands at 1312–1362 cm^−1^ are corresponding to vibrations of C–H and C–C. all of the previously referred peaks are characteristic for cellulosic backbone of cotton fabric. After treatment with either silver or palladium salt precursors, and after dyeing, no significant changing in the estimated characteristic bands of cotton was observed, meaning that, neither dyeing nor implantation of metal particles were affected on the chemical composition of cotton fabrics. However, one new band characteristic for O–M (871 cm^−1^) was visibly noted in the spectral data of all the prepared samples with either silver or palladium. This could declare that, the reducible functional groups for either cellulosic skeleton (for the undyed fabrics) or the phenolic groups of RPN dye moieties (for the dyed fabrics) were successively shared in chemical bonding and stabilization of metal particles.

### XRD analysis

**Figure 4:**
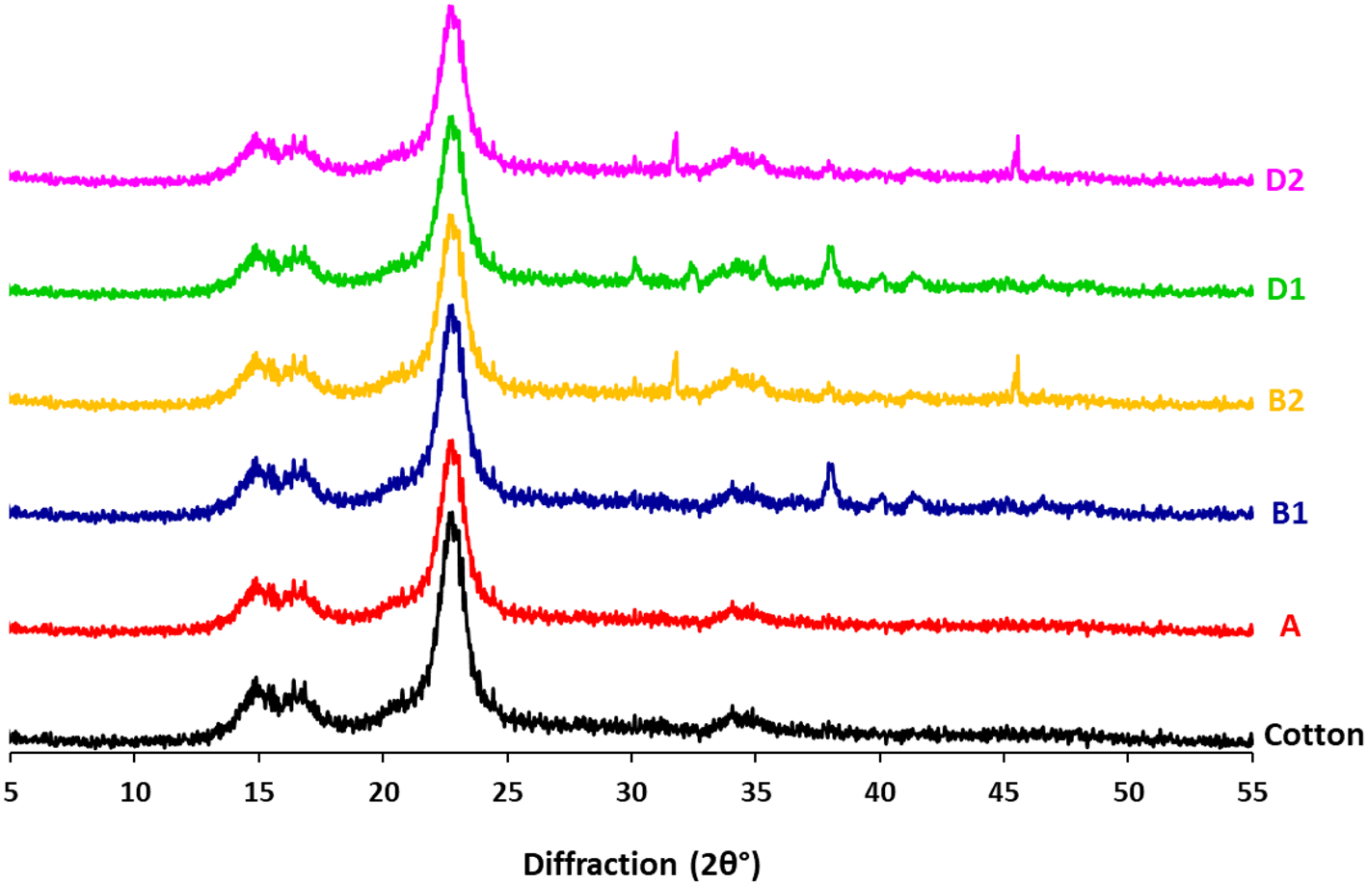
X-ray diffractions for the modified cotton fabrics.

Figure 4 shows XRD data for pristine cotton, dyed cotton (A), undyed (B1 & B2) and dyed fabrics after treatment with silver and palladium precursors (D1 & D2). From the plotted data it could be declared that, untreated cotton fabric exhibited with characteristic diffraction peaks at (2θ°) = 15.1°, 16.9°, 23.2° & 34.2°, referring to the crystalline structure of cellulose in the untreated cotton fabric. Neither dyeing nor successive implantation of nanosilver and nanopalladium particles were affected on the fabric crystallinity, as the as-mentioned characteristic diffraction peaks of cotton were similarly detected for the treated fabrics. After treatment with silver precursor in both of B1 & D1 fabrics, characteristic peak of silver was estimated at (2θ°) = 38.1°, typical for (111) of face center crystalline (FCC) structure for silver (JCPDS data number 04–0783 card^46,47^. Moreover, the fabrics treated with palladium salt precursor were assigned at 2θ = 45.5°, which is corresponded to (200) of face centered crystalline (FCC) structures for Pd (JCPDS data number 89–4897 card)^48,49^.

### XPS analysis

**Figure 5:**
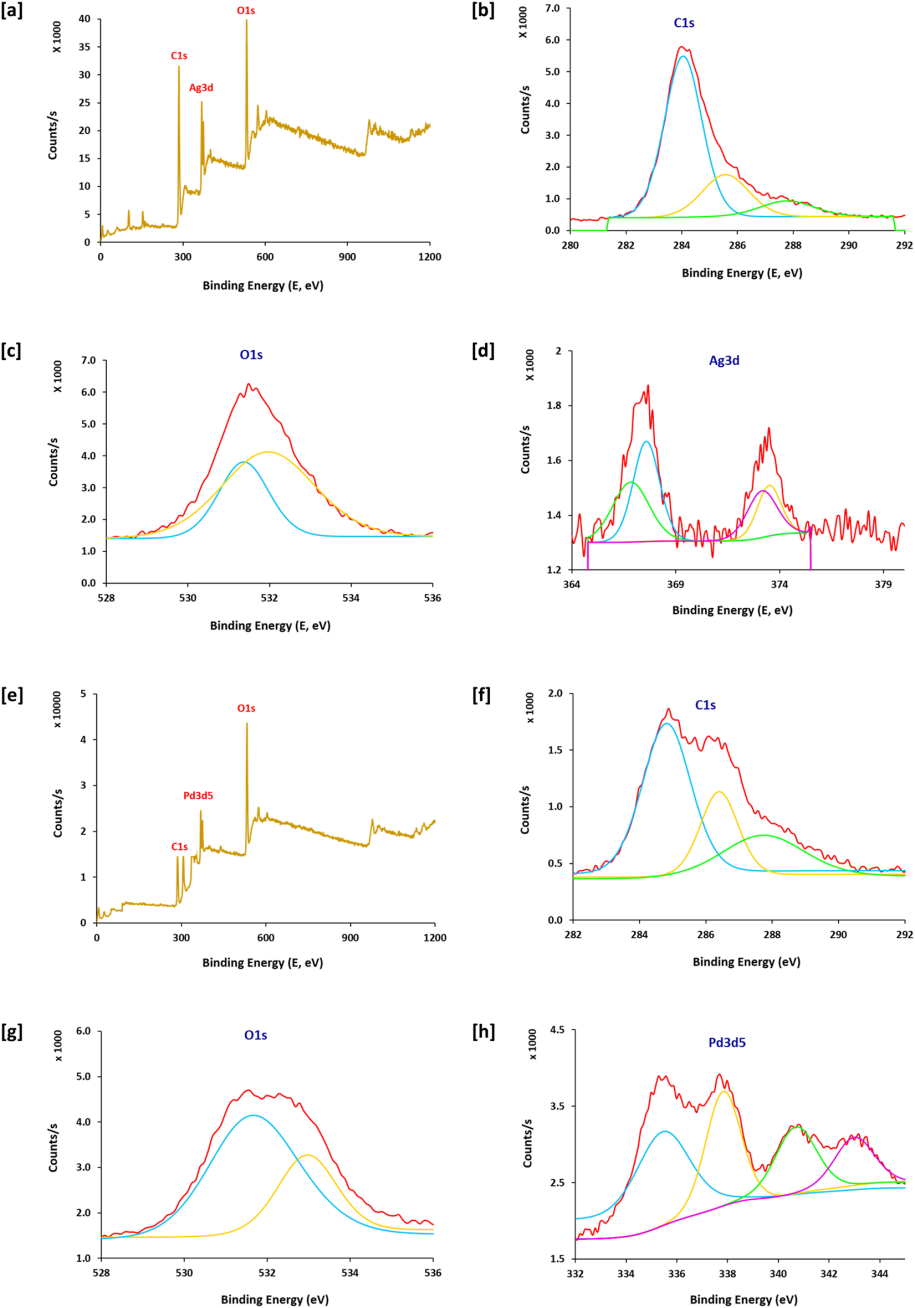
XPS analysis for the modified cotton fabrics; (**a**–**d**) E1 and (**e**–**h**) E2.

XPS spectral mapping data for samples prepared via pre-treatment process (E1 & E2) in Fig. 5 could provide further confirmation about the chemical composition and ionic state for both silver and palladium moieties and its successive immobilization within dyed fabric matrix. The survey spectrum (Fig. 5a and e) could show that, binding energies of C1s, O1s and Ag3d, were located at 281–291, 529–535, and 364–376 eV^50^. C1s bands were estimated at binding energies of 284.1, 285.6 and 288.2 eV that were referred to C–C, C–H and C–O, respectively (Fig. 5b and f)^51^. For O1s, binding energies peaks at 531.3 and 532.1 eV, were corresponding to C–O and C–O–O, respectively (Fig. 5c and g). In case of Ag3d, the bands detected at 367.0 and 373.2/373.6 eV were assigned to 3d_3/2_ and 3d_5/2_ of the metallic form of Ag (Ag^0^) which clearly approved the successive anchoring of silver within fabric matrix (Fig. 5d). Whereas, in case of Pd3d, energy bands detected at 335.5, and 340.8/343.1 eV were referred to 3d_3/2_ and 3d_5/2_ of palladium (Pd^0^) which also affirmed the immobilization of Pd within dyed fabric matrix (Fig. 5h)^52–55^. In Accordance to literature^55–57^, the peaks at 367.7 eV & 337.9 eV were corresponded to the 3d_5/2_ of Ag–O & Pd–O to affirm the coordinative bonding between the immobilized metals and the phenolic groups of RPN dye moieties uploaded within the matrix of the tested fabrics.

### Absorbance spectral mapping

**Figure 6:**
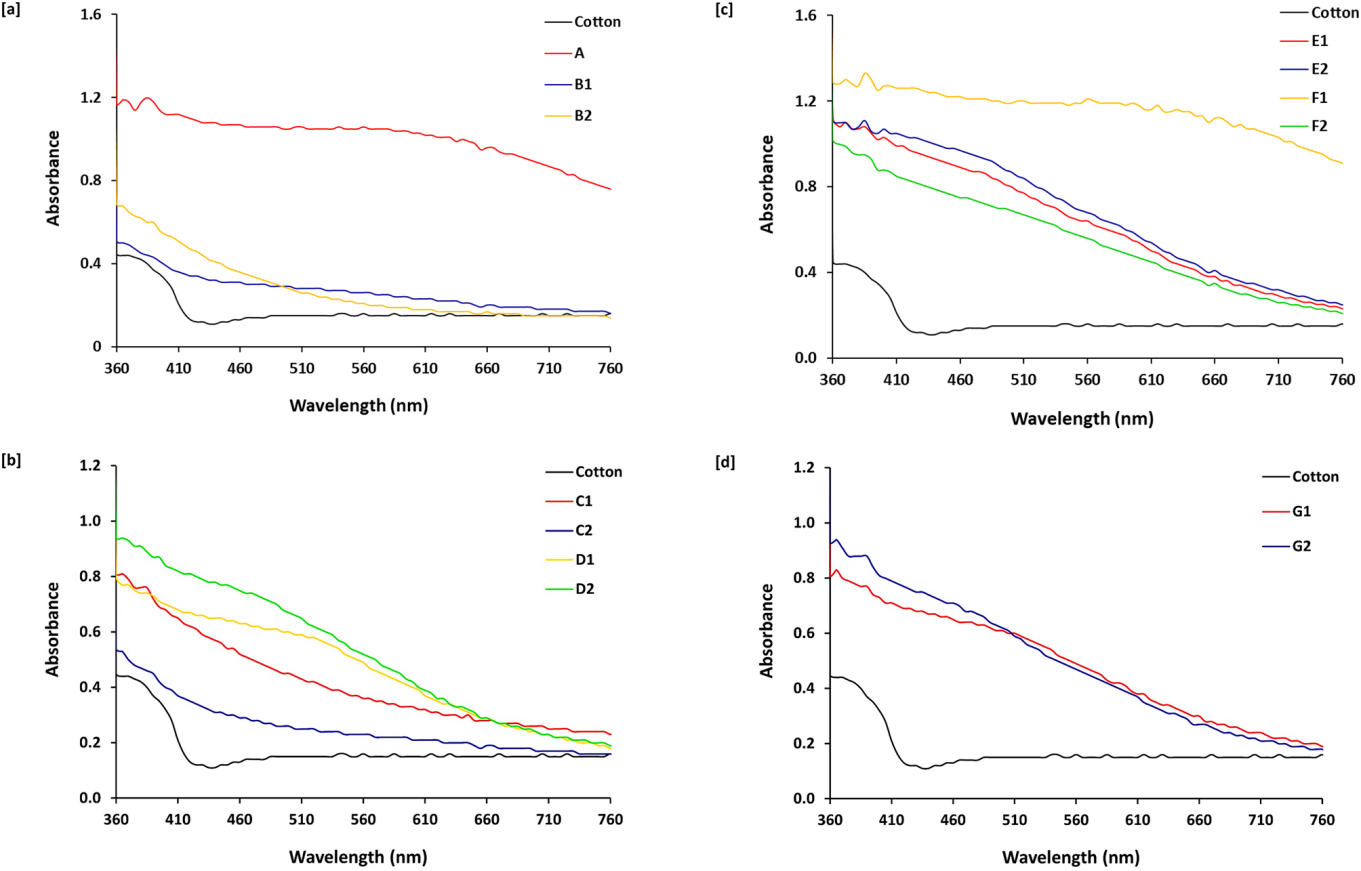
Absorbance for the modified cotton fabrics.

For investigation the effect of metal implantation and the process sequencing on the optical properties of the dyed fabrics, all of the as-prepared samples were analyzed with UV–Visible spectroscopy, and the estimated spectral mapping data were plotted in Fig. 6. From the displayed UV- Visible spectra, the examined samples could be ordered according to the estimated values of intensity as follows; F1 >  > A > E1 ~ E2 ~ D2 ~ F2 ~ G2 >  > C1 ~ G1 ~ D1 ~ B1 ~ B2 ~ C2. This could be explained in the following points, (i) compared to all of the prepared samples, implantation of silver particles within fabric prior to dyeing in the presence of mordant resulted in obtaining a sample with the highest intense color to reflect the role of silver particles for enhancement of fabric coloration, (ii) both of silver and palladium particles could act the role of mordant when the samples were treated with metal precursors prior to dyeing (E1 &E2), (iii) in the presence of dye and regardless to the reaction sequencing, treatment of samples with palladium precursor resulted in obtaining samples with higher intensity values rather than that treated with silver, owing to the higher reactivity of palladium in the presence of dye moieties, however, (iv) in the absence of either dye or mordant, sample treated with silver precursor at 60 °C (sample C1) exhibited with color intensity close to that of dyed sample (A) rather than that treated with palladium, attributing to that, palladium required higher temperature to be more accessible for interacting with fabric backbone.

### Color measurements

**Figure 7:**
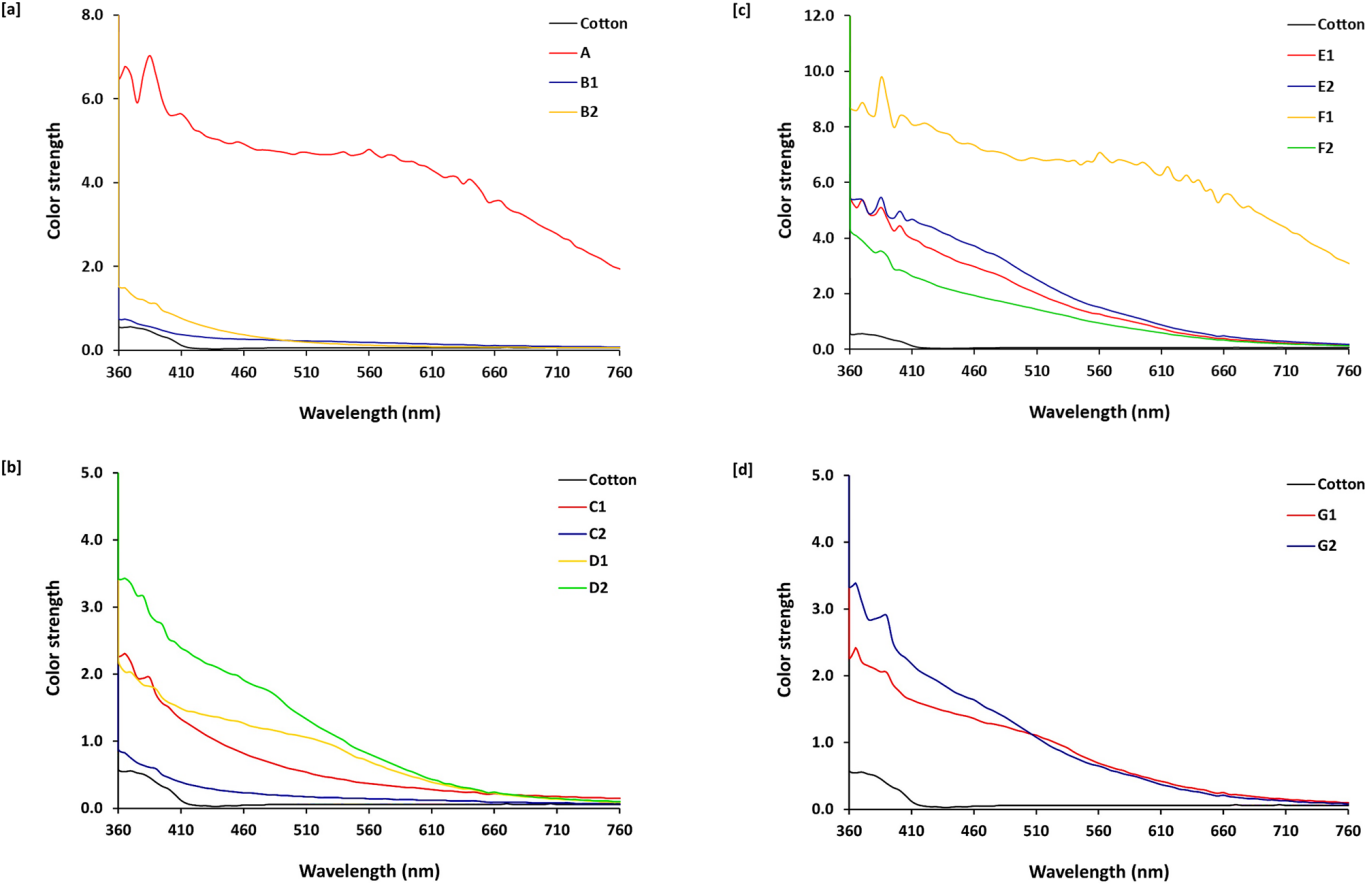
Color strength for the modified cotton fabrics.

In order to approve the effect of metal incorporation on fabric coloration; color coordinates (L*, a* and b*) and color strength (K/S) were evaluated for all of the prepared samples and the data were tabulated in Table 2. From the presented data, it could be recognized that; metal incorporation into the dyed fabric, resulted in significant increment for both of a* and b* positive values, meaning the color changed to the reddish-yellow, attributing to the successive coordination and stabilization of metals^58–63^. Figure 7 also showed the effect of process sequencing on the color strength (*K/S*) to affirm that, samples prepared via pre-treatment process in the absence of mordant (E1 & E2) were observed with color strength values closer to the that of the dyed sample with mordanting (A). Additionally, sample prepared with silver via pre-treatment process in the presence of mordant is exhibited with significantly higher color strength (F1) compared to the dyed cotton (A).

**Table 2:** Color measurement data for the functionalized cotton samples.

Samples	L*	a*	b*	C*	h	Photographical images
Cotton	81.93	0.54	18.56	18.56	88.35	
A	35.98	1.15	1.91	2.23	58.96	
B1	78.97	2.31	6.51	6.91	70.49	
B2	81.59	0.67	18.82	18.84	87.95	
C1	70.71	2.82	17.42	17.65	80.80	
C2	80.88	0.46	8.48	8.50	86.90	
D1	63.62	10.91	14.8	18.39	53.61	
D2	61.35	11.79	21.66	24.66	61.44	
E1	54.90	11.13	22.24	24.87	63.42	
E2	52.30	12.20	23.12	26.14	62.17	
F1	30.60	0.43	2.38	2.42	79.79	
F2	59.20	9.71	18.59	20.97	62.42	
G1	63.26	10.35	16.04	19.09	57.16	
G2	64.06	9.62	22.25	24.24	66.62

Moreover, from the tabulated data in Table 2, it could be declared that, regardless to the reaction conditions, treatment of cotton fabrics with silver precursor resulted in significant increment of reddish color (a*), whereas, yellowness (b*) is shown to visibly higher for samples treated with palladium precursor. Samples that where prepared via pre-treatment process without mordant (E1 & E2) is shown with much higher redness and yellowness compared to the dyed cotton (A). Insignificant differences in the values of a* and b* could be observed, by comparing between the samples prepared via pre-treatment process in the absence (E1 &E2) and in the presence of mordant (F1 &F2). Comparing between the samples prepared via pre-treatment, post treatment and simultaneous processes, the highest values of a* & b* were estimated for samples of prepared via pre-treatment process in the absence of mordant, to signify the accessibility of silver and palladium in playing the role of mordant in fabric coloration. These findings are in harmony with the previously illustrated data.

### Fastness properties

Table 3 represented the estimated values for following up the effect of reaction sequencing and coordinative clustering and stabilization of metals on the fastness properties of the currently-prepared fabrics. Data revealed that, regardless to the reaction sequencing, the successive coordination of metals either silver or palladium resulted in significant enhancement in the fastness properties. From the estimated data it could depict that, successive immobilization of metal precursors using the currently demonstrated techniques did not affect on rubbing and washing fastness and significantly enhanced the light fastness. Samples that prepared via the pre-treatment process in absence of mordant (E1 & E2) were shown good rubbing and excellent washing fastness, while, light fastness was graded as outstanding. So, in summarization, either silver or palladium particles were successfully played the role of mordant for chemical stabilization of dye moieties within the fabric matrix.

**Table 3:** Fastness properties for the functionalized cotton samples.

Sample	Rubbing test		Light	Wash
	Dry	Wet		
A1	3	3	5	5
B1	3–4	3	5	4–5
B2	3	3	5	4–5
C1	3	3	5	4–5
C2	3–4	3–4	4–5	4–5
D1	4	3–4	5	5
D2	3–4	3–4	5	5
E1	3–4	3–4	5–6	4–5
E2	3	3	5–6	5
F1	3–4	3–4	6	5
F2	3	3	6	4–5
G1	3	3–4	5	4–5
G2	3–4	3–4	4–5	5

### UV-blocking potentiality

Cotton textiles were ascribed as one of the highly applicable textile materials in various purposes, attributing to their biocompatibility, smoothness, and breathability, however, it could exhibit some of disadvantages such as prone to high flammability, biological attacking, and low ultraviolet resistance^9–11^. Additionally, protective textile materials must be characterized with high UV ray reflecting and/or absorption affinity so as it could prevent UV irradiation from threatening the human body.

Therefore, recent reports were considered with preparation of UV-protective textiles. Numerous systematic studies were performed for exploiting some of metallic oxides (like, titanium dioxide, silicon dioxide, zirconium dioxide, magnesium dioxide and zinc oxide), and some of organic polymers were successively applied for treatment of cotton-based textiles to acquire the prepared textiles special properties^64–66^. In the current study, an innovative approach was demonstrated for acquirement the treated cotton fabric with UV-protective potency via successive immobilizing of silver and palladium within cotton dyed with RPN dye.

**Figure 8:**
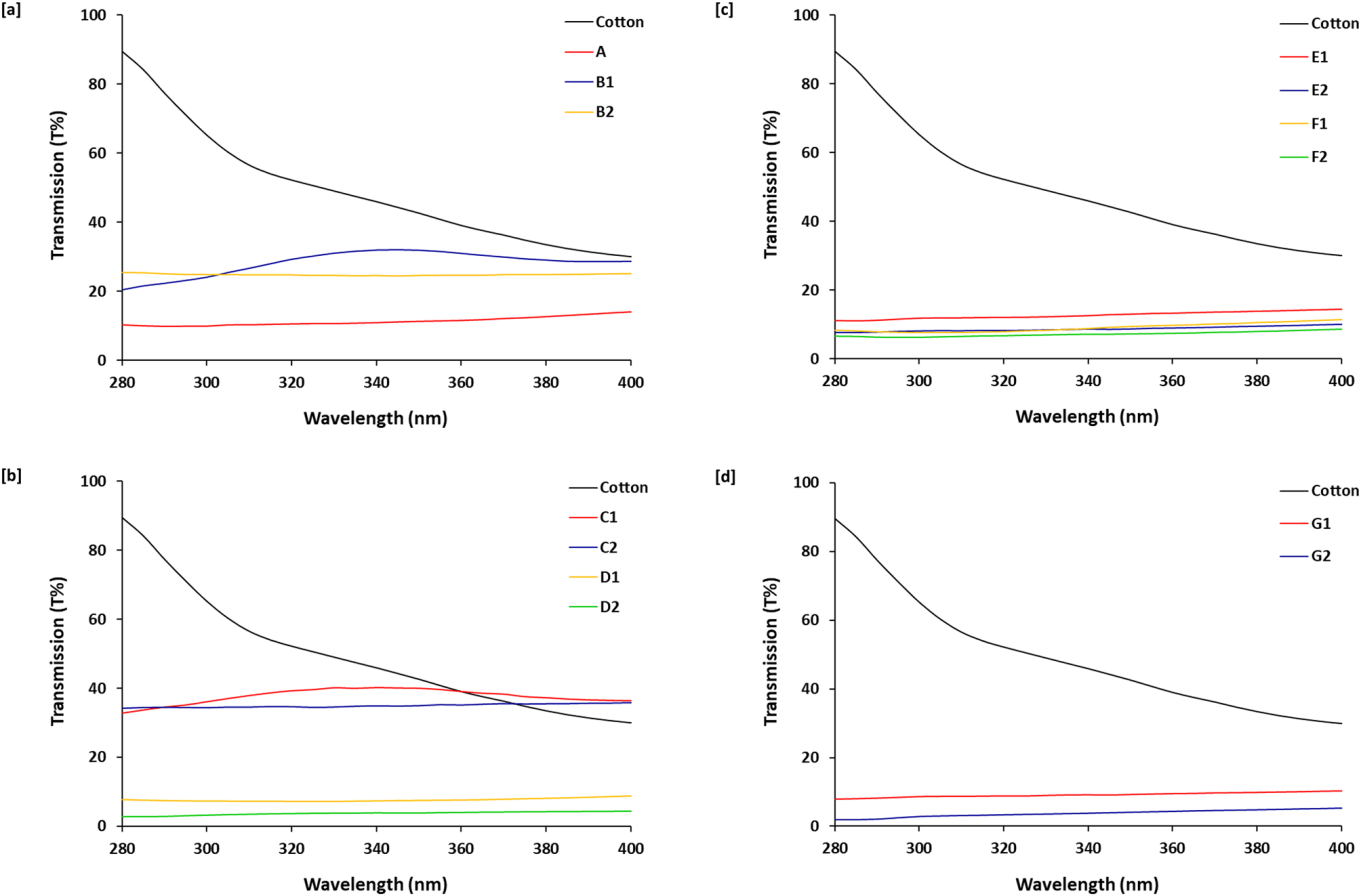
Transmission radiation through the modified cotton fabrics.

Transmission percent (T%, Fig. 8) and Ultraviolet Protection Factor (UPF, Table 4) could be described as key parameters for evaluating the ultraviolet blocking affinity for all of the currently prepared samples. According to the tabulated data, it could be revealed that, the blocking percent of treated samples showed that UV-B blocking percent is significantly higher than that for UV-A for all samples. Ultraviolet blocking potency was analyzed in a wavelength range of 280−400 nm, as shown in Fig. 8, for all of the prepared samples. T% of cotton fabric was estimated to be ca. 58.2% and 99.8% for both of UV-A and UV-B, respectively. As demonstrated in Fig. 8, the protection percentage for all of the prepared fabrics indicated with minor UV transmittance compared to dyed sample, i.e., T% of all the prepared samples was observably diminished after immobilization of metal particles within dyed samples.

**Table 4:** Ultraviolet protection results for the prepared samples.

Sample	UPF	UVA (T%)	UVB (T%)	UVA blocking	UVB blocking
Cotton	1.2	58.2	99.8	41.8	0.2
A1	9.5	11.9	10.2	88.1	89.8
B1	3.7	30.2	24.0	69.8	76.0
B2	4.0	24.7	25.0	75.3	75.0
C1	2.6	38.6	35.8	61.4	64.2
C2	2.9	35.2	34.6	64.8	65.4
D1	13.7	7.7	7.4	92.3	92.6
D2	28.6	4.0	3.1	96.0	96.9
E1	8.3	13.1	11.5	86.9	88.5
E2	12.0	9.0	8.0	91.0	92.0
F1	12.8	9.5	7.8	90.5	92.2
F2	15.2	7.5	6.5	92.5	93.5
G1	11.3	9.5	8.5	90.5	91.5
G2	31.5	4.2	2.6	95.8	97.4

Moreover, the presented data in Table 4 show that, regardless to the reaction sequencing, samples that were dyed using either silver or palladium instead of mordant were exhibited with higher UPF, UVA blocking and UVB blocking values compared to that dyed with mordanting (A). However, samples that were prepared with palladium salt were exhibited with significantly higher values of UPF rather than that estimated for samples prepared with silver precursor. In the absence of mordant, samples prepared with post-treatment process (D2, UPF 28.6, UVB T% 3.1%, UVB blocking percent 96.9%) exhibited with higher UV-resistance rather than that prepared with pre-treatment process (E2, UPF 12.0, UVB T% 8.0%, UVB blocking percent 92.0%). However, the sample prepared with simultaneous process showed with the highest UV-blocking action (G2, UPF 31.5, UVB T% 2.6%, UVB blocking percent 97.4%). However, it could be decided that, whatever the applied process for sample treatment, exploitation of either silver or palladium instead of mordant acquired the dyed samples excellent UV-blocking potentiality.

### Antimicrobial action

Investigative approaches concerned in biotechnology spurs extensive consideration for manufacturing of multi-functional textiles^16,18,19,67–69^. According to literature^18,62,70^ silver precursors were exploited in manufacturing of biologically active textiles, whereas, no previous reports were studied the antimicrobial activities of dyed cotton immobilized with the as-applied metal precursors. In the current approach, the leadership role of the immobilized metals for dyeing and functionalization of the treated fabrics to be efficiently applicable in medical purpose is approved. Herein, the antimicrobial potency for the treated fabrics was evaluated against *E. coli* (G –ve bacteria), *S. aureus* (G + ve bacteria) and *Candida albicans* through inhibition zone and the reduction percent was estimated to be tabulated in Table 5. The estimated data revealed that, against *E. coli*, *S. aureus* and *Candida albicans*, respectively, comparing to the dyed sample with mordanting (A, 65.52%, 74.90% & 58.00%), only treatment of samples with either silver (B1, 84.04%, 89.21% & 85.96%) or palladium (B2, 80.89%, 86.21% & 79.58%) acquired the samples very good antimicrobial activities. However, the samples prepared via pre-treatment process in absence of mordant showed excellent antimicrobial action by exploiting either silver (E1, 89.56%, 96.71% & 93.01%) or palladium (E2, 92.17%,89.51% & 92.87C%). So, it could be eventually decided that, successive implantation of either silver or palladium showed to act the role of mordant with higher color fastness, in addition to acquire the treated fabrics excellence in UV-protection and antimicrobial actions.

**Table 5:** Reduction percentage of different pathogens for the modified cotton fabrics.

Samples	*Escherichia coli*	*Staphyllococus aureus*	*Candida albicans*
Blank	0.0	0.0	0.0
A	65.52	74.90	58.00
B1	84.04	89.21	85.96
B2	80.89	86.21	79.58
E1	89.56	96.71	93.01
E2	92.17	89.51	92.78

## Conclusions

In the represented approach, a systematic study is demonstrated for approving the superior role of silver and palladium metallic particles to act the role of mordant, with enhancing the color fastness and acquiring the dyed (with extract of red peanuts skin) cotton fabrics excellent antimicrobial potentiality and UV-protective affinity. From the represented data it could be declared that, the samples treated with metal precursors prior to dyeing were exhibited with the highest color strength, very good–excellent color fastness, very good UV blocking (UVB blocking percent was 97.4), and excellence biocidal potency (microbial reduction percent was ranged in 93.01–99.51%).

## Data Availability

The all data generated or analyzed during the current study are included in this manuscript.
