# Author Correction: Involvement of silver and palladium with red peanuts skin extract for cotton functionalization

**DOI:** 10.1038/s41598-024-60771-7

**Published:** 2024-05-01

**Authors:** Hossam E. Emam, Nancy S. El-Hawary, Hamada M. Mashaly, Hanan B. Ahmed

**Affiliations:** 1 Pre-treatment and Finishing of Cellulosic Fibers, Textile Research and Technology Institute, National Research Centre, 33 EL Buhouth St., Dokki, Giza, 12311 Egypt; 2 Dyeing, Printing and Auxiliaries Department, Textile Research and Technology Institute, National Research Centre, 33 EL Buhouth St., Dokki, Giza, 12311 Egypt; 3 Chemistry Department, Faculty of Science, Helwan University, Ain-Helwan, Cairo, 11795 Egypt

Correction to: *Scientific Reports* 10.1038/s41598-023-43267-8, published online 26 September 2023

In the original version of this Article Hossam E. Emam was incorrectly affiliated with ‘Chemistry Department, Faculty of Science, Helwan University, Ain-Helwan, Cairo, 11795, Egypt’. Their correct affiliation is listed below.

Pre-treatment and Finishing of Cellulosic Fibers, Textile Research and Technology Institute, National Research Centre, 33 EL Buhouth St., Dokki, Giza, 12311, Egypt.

The original Article, as well as the previously published Author Correction have been corrected.

